# β-Lactam Effects on Mixed Cultures of Common Respiratory Isolates as an Approach to Treatment Effects on Nasopharyngeal Bacterial Population Dynamics

**DOI:** 10.1371/journal.pone.0003846

**Published:** 2008-12-04

**Authors:** David Sevillano, Lorenzo Aguilar, Luis Alou, María-José Giménez, Natalia González, Martha Torrico, Fabio Cafini, Pilar Coronel, José Prieto

**Affiliations:** 1 Microbiology Department., School of Medicine, University Complutense, Madrid, Spain; 2 Scientific Department, Tedec-Meiji Farma S. A., Madrid, Spain; University College London, United Kingdom

## Abstract

**Background:**

*Streptococcus pneumoniae*, *Streptococcus pyogenes* and *Haemophilus influenzae* are bacteria present in the nasopharynx as part of normal flora. The ecological equilibrium in the nasopharynx can be disrupted by the presence of antibiotics.

**Methodology/Principal Findings:**

A computerized two-compartment pharmacodynamic model was used to explore β-lactam effects on the evolution over time of a bacterial load containing common pharyngeal isolates by simulating free serum concentrations obtained with amoxicillin (AMX) 875 mg tid, amoxicillin/clavulanic acid (AMC) 875/125 mg tid and cefditoren (CDN) 400 mg bid regimens over 24 h. Strains and MICs (µg/ml) of AMX, AMC and CDN were: *S. pyogenes* (0.03, 0.03 and 0.015), *S. pneumoniae* (2, 2 and 0.25), a β-lactamase positive *H. influenzae* (BL^+^; >16, 2 and 0.06) and a β-lactamase positive AMC-resistant *H. influenzae* (BLPACR, >16, 8 and 0.06). Mixture of identical 1∶1∶1∶1 volumes of each bacterial suspension were prepared yielding an inocula of ≈4×10^6^ cfu/ml. Antibiotic concentrations were measured both in bacterial and in bacteria-free antibiotic simulations. β-lactamase production decreased AMX concentrations and *f*T_>MIC_ against *S. pneumoniae* (from 43.2% to 17.7%) or *S. pyogenes* (from 99.9% to 24.9%), and eradication was precluded. The presence of clavulanic acid countered this effect of co-pathogenicity, and *S. pyogenes* (but not BL^+^ and *S. pneumoniae*) was eradicated. Resistance of CDN to TEM β-lactamase avoided this co-pathogenicity effect, and CDN eradicated *S. pyogenes* and *H. influenzae* strains (*f*T_>MIC_ >58%), and reduced in 94% *S. pneumoniae* counts (*f*T_>MIC_ ≈25%).

**Conclusions/Significance:**

Co-pathogenicity seems to be gradual since clavulanic acid countered this effect for strains very susceptible to AMX as *S. pyogenes* but not for strains with AMX MIC values in the limit of susceptibility as *S. pneumoniae*. There is a potential therapeutic advantage for β-lactamase resistant cephalosporins with high activity against streptococci.

## Introduction

Carriage of common respiratory isolates as *Haemophilus influenzae*, *Streptococcus pneumoniae* and *Streptococcus pyogenes* depends on multiple factors such as active or passive smoking, crowding or age [1], strain fitness properties [2], specific vaccination [3], and bacterial interference in antibiotic-free niches. *H. influenzae* and *S. pneumoniae* are recovered exclusively from humans and find their ecological niche in colonizing nasopharynx. Up to 80% of healthy persons carry *H. influenzae* [4], with multiple strains in 50% positive samples [5]. In the case of *S. pneumoniae*, carriage ranges from 10% to 40% in an age dependent manner [6], with a lower percentage of multiple strains in the same sample [7]. While higher turnover of strains is found in *H. influenzae* [2], duration of nasopharyngeal carriage of penicillin-resistant streptococci (PRSP) depends on age, seasonality, carriage of PRSP by other family members [8], and serotype, with higher transmission for 6A and 14 [9]. The carriage of strains resistant to β-lactams is a source of concern in some countries as Spain where penicillin non-susceptibility in *S. pneumoniae* reaches 44% isolates in the community [10], and ampicillin resistance in *H. influenzae* attains 25%, with 80% of these isolates being resistant due to β-lactamase production and 20% due to the BLNAR (β-lactamase negative ampicillin-resistant) phenotype [10] caused by mutations in the *fts*I gene [11]. Strains exhibiting both resistant genotypes (TEM- β-lactamase and mutation in the *fts*I gene) constitute the BLPACR (β-lactamase positive amoxicillin/clavulanate-resistant) phenotype and have been recently reported as cause of concern [11].

No problems of resistance to β-lactams are found in *S. pyogenes*, an ubiquitous microorganism that frequently colonizes throats of asymptomatic persons, with carriage rates of 15–20% in infants [12], [13] and of 10% in adult smokers [1].

Mucosal surfaces may be simultaneously colonized by multiple species, and there is an intrincate balance in the oropharynx between *S. pyogenes*, *H. influenzae*, *S. pneumoniae* and other oropharyngeal flora [14]. It has been suggested that this oropharyngeal flora is altered in *S. pyogenes* carriers with a decrease in other streptococcal species (alpha-hemolytic streptococci) with interfering capabilities [14], [15]. On the other side the success of an organism in colonizing, and maybe in establishing a subsequent infection, might be determined by its ability to compete with co-habitants of its niche, since replication/survival of biological organisms serves to gain space and time [16], and to gain space is dominance (versus other populations) and to gain time is success [16]. Dynamics of different bacterial populations in antibiotic-free niches are the baseline that antibiotic treatments can alter [17], [18].

In this study we explored the effect of physiological concentrations of three β-lactams (amoxicillin, amoxicillin plus a β-lactamase inhibitor, and a third generation oral cephalosporin resistant to TEM β-lactamases) on the evolution over time of a bacterial load containing a beta-hemolytic streptococci (*S. pyogenes*), an alpha-hemolytic streptococci (a serotype 14 penicillin-resistant *S. pneumoniae*), and two β-lactamase positive *H. influenzae* strains (one characterized as BLPACR) in the same niche.

## Materials and Methods

### Strains

Four clinical isolates were used throughout the study: one *S. pyogenes*, one serotype 14 penicillin-resistant *S. pneumoniae*, one β-lactamase TEM-1 producing *H. influenzae* (BL^+^), and one *H. influenzae* TEM-1 positive strain presenting a N526K mutation in the *fts*I gene (BLPACR). BL^+^ was trimethoprim resistant (MIC >128 µg/ml) and the BLPACR strain was trimethoprim susceptible (0.12 µg/ml); this difference was used throughout the study to differentiate both strains. Mutations in the *fts*I and TEM-1 genes were determined by PCR amplification and direct sequencing [19], [20].

### Culture media and preliminary studies

Different broth media were used in preliminary studies to determine which media showed the best growth rate over 24 h for all strains: a) Mueller-Hinton broth (Difco laboratories, Detroit, Mich.) supplemented with 5% lysed sheep blood (Biomedics, Madrid, Spain) (MHB) (as media recommended for streptococci) [21], b) Mueller-Hinton broth supplemented with 15 µg/ml nicotinamide adenine dinucleotide, 15 µg/ml haemin (Sigma-Aldrich Chemical Co., St. Louis, USA), and 5 µg/ml of yeast extract (Difco laboratories) (HTM) (as media recommended for *H. influenzae*) [21], c) Todd-Hewitt broth supplemented with 5 µg/ml of yeast extract, and d) Todd-Hewitt broth (Difco laboratories) supplemented with 15 µg/ml nicotinamide adenine dinucleotide, 15 µg/ml haemin and 5 µg/ml of yeast extract (THSB). THSB showed the best growth rate for all strains (data not shown) and was the selected broth medium for pharmacodynamic simulations.

### In vitro susceptibility

MICs of cefditoren, amoxicillin and amoxicillin/clavulanic acid were determined by the microdilution method following NCCLS/CLSI recommendations [21], and also using THSB as media (broth media used in pharmacodynamic simulations). MICs were determined in triplicate and the modal value of each determination was considered.

### In vitro kinetic model *(* *Figure 1* *)*

A previously described two-compartment dynamic model was used to expose bacteria to changing study drug concentrations avoiding the dilution of the bacterial inoculum together with the drug [17], [18]. The extra-capillary space and the intra-dialyser circulating tubing of the second compartment (FX50 helixone dialyzer, Fresenius Medical Care S.A., Barcelona, Spain), represented the colonisation site. The central compartment, representing the systemic circulation, consisted of a spinner flask with THSB, tubing and lumina of capillaries within a dialyser unit. The exponential decay of concentrations was obtained by a continuous dilution-elimination process using computerized peristaltic pumps (Masterflex, Cole-Parmer Instrument Co., Chicago, IL, USA) set to simulate half-lives of amoxicillin, clavulanic acid and cefditoren. In control drug-free simulations the rate of peristaltic pumps was fixed to 0.67 ml/min. Additional pumps circulated the antimicrobial-medium mixture at 50 ml/min rate between the central and peripheral compartments, and at 25 ml/min within the extra-capillary space through external tubing. A computer-controlled syringe pump (402 Dilutor Dispenser; Gilson S.A, Villiers-le-Bel, France) allowed the simulation of drug concentrations by infusion of the drug into the central compartment until the maximum concentration achieved in serum (Cmax) was reached. Both compartments were maintained at 37°C all over the simulation process.

**Figure 1:**
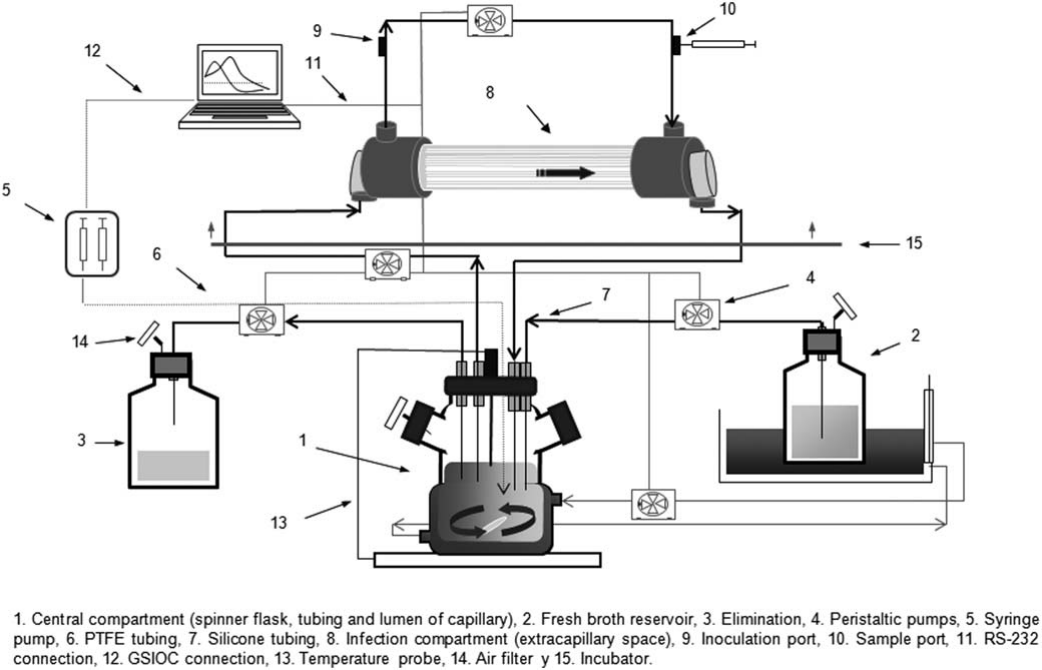
Pharmacodynamic computerized device. Diagram of the in vitro two-compartment computerized device used in the study.

### Preparation of individual and mixed cultures

The strains were grown overnight on Mueller-Hinton agar (Difco laboratories) supplemented with 5% lysed sheep blood (Biomedics) (MHA) in the case of *S. pneumoniae* and *S. pyogenes*, or on GC agar (Difco laboratories) supplemented with 5% sheep blood (added at 50°C) and VX growth factors (GCSA) in the case of *H. influenzae*. Several colonies of each strain were suspended in THSB to obtain a bacterial density of approx. 1×10^8^ cfu/ml. A 1∶100 dilution was prepared to obtain an initial inoculum of approx. 1×10^6^ cfu/ml. For the mixed inoculum, a mixture of identical 1∶1∶1∶1 volumes of each bacterial suspension was prepared, yielding an inoculum of approx. 4×10^6^ cfu/ml.

### Experiments

Sixty ml of each individual or of the mixed inoculum were introduced into the peripheral compartment of the in vitro model and pre-incubated 1 h. Experiments performed were:

- Antibiotic-free simulations using each individual inoculum (individual growth control)
- Antibiotic-free simulations using the mixed inoculum (mixed growth control)
- Bacteria-free simulations with each antibiotic to set the pharmacokinetic profile of each study drug
- Simulations with each antibiotic using the mixed inoculum

In each experiment, samples (0.5 ml) from the peripheral compartment were collected at 0, 2, 4, 6, 8, 10, 12, 24 h. Each sample was serially ten-fold diluted in 0.9% sodium chloride, and 20 µl plated for bacterial counting onto plates containing different media to allow species/strains differentiation: a) MHA: growth of *S. pneumoniae* and *S. pyogenes* that were differentiated by their different hemolytical properties (beta for *S. pyogenes* and alpha for *S. pneumoniae*), b) GCSA: growth of both *H. influenzae* strains that were differentiated from the other species by their morphological properties, c) GCSA containing 4 µg/ml of trimethoprim: growth of BL^+^, not allowing the growth of the BLPACR strain. Colony counts of the BLPACR strain were calculated by the difference in colony counts between both GCSA plates. The total population was established as the accumulated colony counting of *S. pneumoniae* and *S. pyogenes* on MHA and *H. influenzae* on GCSA medium. All plates were incubated at 37°C 5% CO_2_ for 24 h. The limit of detection was 50 cfu/ml. All experiments were performed in triplicate.

### Kinetic simulations

Free-drug concentrations after oral amoxicillin 875 mg three-times daily, amoxicillin/clavulanic acid 875/125 mg three-times daily and cefditoren-pivoxil 400 mg twice daily administration, were simulated over 24 h using the reported protein binding of 18% for amoxicillin [22], 25% for clavulanic acid [22], and 88% for cefditoren [23]. The target total pharmacokinetic parameters were: Cmax = 11.6 µg/ml and t1/2 = 1.15 h for amoxicillin [22], Cmax = 2.20 µg/ml and t1/2 = 1.15 h for clavulanic acid [22], and Cmax = 4.5 µg/ml and t1/2 = 1.55 h for cefditoren [23]. Target free concentrations and pharmacokinetic parameters are shown in Table 1.

**Table 1:** Pharmacokinetics.

	Cefditoren 400 mg			Clavulanic acid 125 mg			Amoxicillin 875 mg				
								AMX simulations		AMC simulations	
Time (h)	Target	S_BF_	S_MI_	Target	S_BF_	S_MI_	Target	S_BF_	S_MI_	S_BF_	S_MI_
Tmax^a^	0.50	0.52±0.02	0.52±0.03	1.65	1.70±0.09	1.46±0.11	9.51	9.05±0.19	5.11±1.27^b^	9.17±0.24	9.24±0.71
2	-	0.36±0.00	0.40±0.04	1.22	1.15±0.13	1.07±0.21	7.03	6.30±0.51	2.43±1.49^b^	6.12±0.09	6.04±0.91
4	0.29	0.28±0.04	0.30±0.02	0.36	0.21±0.03	0.21±0.03	2.11	1.74±0.00	UDL^b^	1.50±0.17	0.59±0.39^b^
6	0.12	0.12±0.01	0.10±0.03	0.11	0.13±0.00	0.11±0.00	0.63	0.45±0.03	UDL^b^	0.41±0.05	0.06±0.02^b^
8	0.04	0.04±0.00	0.05±0.01	0.03	UDL	UDL	0.19	0.13±0.03	UDL^b^	0.17±0.02	0.04±0.01^b^
10	0.02	0.02±0.00	0.01±0.01	-	-	-	-	-	-	-	-
12	0.01	0.01±0.00	0.01±0.00	-	-	-	-	-	-	-	-
Cmax^c^	0.50	0.52±0.02	0.52±0.03	1.65	1.70±0.09	1.46±0.10	9.51	9.06±0.19	5.11±1.27^b^	9.17±0.24	9.24±0.71
AUC^d^	1.79	1.76±0.05	1.79±0.10	3.91	3.26±0.09	2.86±0.40	22.59	20.13±0.52	5.61±1.71^b^	19.76±0.91	15.90±1.59
t1/2 (h)	1.55	1.49±0.06	1.48±0.07	1.15	0.99±0.31	1.03±0.17	1.15	1.08±0.09	0.40±0.06^b^	1.16±0.03	0.80±0.03 b

Free concentrations (in beta-phase over the dosing interval) and pharmacokinetic parameters: target values and values determined in bacteria-free simulations (S_BF_) and in simulations with the mixed inocula (S_MI_).

a Tmax: 2.8 h for cefditoren, 1.5 h for clavulanic acid and amoxicillin.

b p<0.01 versus S_BF_.

c µg/ml.

d mg×h/L.

UDL = under detection limit.

### Pharmacokinetic analysis

For the measurement of simulated antimicrobial concentrations, aliquots (0.5 ml) were taken at 0, 2, 4, 6, 8, 10, 12 and 24 h and at the time corresponding to Tmax, and stored at −50°C. To study the influence of bacteria on the pharmacokinetic profile, samples for measuring antibiotic concentrations were taken both in bacteria-free antibiotic simulations (carried out to set the model) and in experimental simulations with bacteria and antibiotic. Concentrations were determined by bioassay using *Morganella morganii* ATCC 8076H as indicator organism for cefditoren [24] (linear concentrations from 0.0035 to 4 µg/ml; limit of detection = 0.0035 µg/ml), *Micrococcus luteus* ATCC 9341 for amoxicillin (linear concentrations from 0.03 to 1 µg/ml; limit of detection = 0.06 µg/ml), and *Klebsiella pneumoniae* NCTC 11228 for clavulanic acid concentrations [25] (linear concentrations from 0.06 to 4 µg/ml; limit of detection = 0.12 µg/ml). Plates were inoculated with an even lawn of the indicator organism and incubated for 18–24 h at 37°C. Intra- and inter-day coefficients of variation were 2.54% and 2.25% for amoxicillin, 3.68% and 2.02% for clavulanic acid, and 1.97% and 1.97% for cefditoren, respectively, for an internal control concentration of 0.75 µg/ml.

Antimicrobial concentrations were analysed by a non-compartmental approach using WinNonlin 5.2 Professional program (Pharsight, Mountainview, CA, USA). Cmax and Tmax were obtained directly from observed data and the area under the concentration-time curve (AUC) was calculated by the trapezoidal rule. The percentage of the dosing interval that the unbound fraction of drug concentrations exceed the MIC, *f*T_>MIC_, was calculated by a non-comportmental approach for pharmacodynamic data using the model 220 of WinNonlin program.

### Measurement of β-lactamase activity

β-lactamase activity was measured in antibiotic simulations with the mixed inocula at 0, Tmax, 2, 4, 6, 8, 10, 12 and 24 h using a modification of a previously described method [26], [27]. In brief, 0.025 ml of a 500 µg/ml solution of Nitrocefin were added to 225 µl of samples collected at sampling times for colony counting, and incubated for 30 min at 37°C. Afterwards, 0.75 ml of phosphate buffer 0.05 M was added and absorbance at 486 nm (A_486_) was spectrophotometrically read, using broth without inoculum as baseline absorbance.

### Statistical analysis

Unpaired t test or one-way ANOVA with Tukey post test was used to compare the concentrations measured and the pharmacokinetic parameters calculated in bacteria-free simulations vs. simulations with the mixed inocula for each study drug, and of β-lactamase production between simulations with the different study drugs. A p≤0.01 was considered statistically significant.

## Results

### Pharmacokinetics

Table 1 shows concentrations and pharmacokinetic parameters experimentally measured in the peripheral compartment in bacteria-free simulations and in those simulations performed with the mixed inocula. For cefditoren and clavulanic acid, no significant differences were found between target free concentrations, those determined in bacteria-free simulations and those in mixed inocula simulations. In contrast, for amoxicillin significant differences were found between concentrations determined in simulations carried out with the mixed inocula and those measured in bacteria-free simulations. In amoxicillin simulations (where amoxicillin was not protected by clavulanic acid) significant lower concentrations of amoxicillin were found in simulations carried out with the mixed inocula from Tmax to 8 h (end of the dosing interval), with non-detectable amoxicillin concentrations from 4 h on. This resulted in a significant decrease in amoxicillin pharmacokinetic parameters calculated with amoxicillin concentrations measured in simulations carried out with the mixed inocula (with its β-lactamase production) vs. those calculated with concentrations measured in bacteria-free simulations: Cmax decreased from 9.06 to 5.11 µg/ml, AUC from 20.13 to 5.61 µg/ml×h, and t1/2 from 1.08 to 0.40 h. In amoxicillin/clavulanic acid simulations, regardless the presence of clavulanic acid, significant lower concentrations of amoxicillin were found in simulations carried out with the mixed inocula from 4 to 8 h, although differences in pharmacokinetic parameters were not significant except for the half-life value (1.16 vs. 0.80 h).

### In vitro susceptibility

Table 2 shows in vitro susceptibility (MICs determined following CLSI recommendations) of study strains to the antibiotics used in this study. MICs determined in THSB (the broth media used in the simulations) exhibited equal values to those determined in the media recommended by CLSI, except for the BLPACR strain and cefditoren, and the BL^+^ strain and amoxicillin/clavulanic acid where MICs in THSB showed one-dilution lower values. *S. pyogenes* was susceptible to all antibiotics with MICs ≤0.03 µg/ml. *S. pneumoniae* was susceptible to amoxicillin with or without clavulanic acid (MIC = 2 µg/ml), and although there are not established CLSI breakpoints for cefditoren, the strain would be classified as intermediate resistant (MIC = 0.25 µg/ml) following the FDA proposed breakpoints [23]. Both *H. influenzae* strains were inhibited by cefditoren concentrations of 0.06 µg/ml, were resistant to amoxicillin, and the BLPACR strain was also resistant to amoxicillin/clavulanic acid according to CLSI breakpoints [28].

**Table 2:** Pharmacodynamics.

	Cefditoren			Amoxicillin			Amoxicillin/clavulanic acid		
	MIC	T>MIC		MIC	T>MIC		MIC	T>MIC^a^	
		S_BF_	S_MI_		S_BF_	S_MI_		S_BF_	S_MI_
*S. pyogenes*	0.015	84.3	79.0	0.03	99.9	24.9	0.03	99.9	99.9
*S. pneumoniae*	0.25	24.0	25.6	2	43.2	17.7	2	40.8	32.8
*H. influenzae* BL^+^	0.06	57.4	58.0	>16	0.0	0.0	2	40.8	32.8
*H. influenzae* BLPACR	0.06	57.4	58.0	>16	0.0	0.0	8	4.5	4.6

In vitro susceptibility (MIC; µg/ml) of the strains used in the study, and T>MIC (% dosing interval) in bacteria-free simulations (S_BF_) and in simulations with the mixed inocula (S_MI_) for bid 400 mg cefditoren, tid 875 mg amoxicillin, and tid 875/125 mg amoxicillin/clavulanic acid regimens.

a on amoxicillin basis.

### Pharmacodynamics

Table 2 shows *f*T_>MIC_ calculated both with concentrations determined in bacteria-free simulations and in those with the mixed inocula. Similar values were obtained in both cases for cefditoren with *f*T_>MIC_ >55% for *H. influenzae* and *S. pyogenes*, and ≈25% for *S. pneumoniae*. In the case of amoxicillin/clavulanic acid, negligible values of amoxicillin *f*T_>MIC_ were obtained against the BLPACR strain, values for the BL^+^ and *S. pneumoniae* decreased from 40.8% to 32.8% when comparing bacteria-free vs. mixed inocula (with its β-lactamase production), and were nearly 100% of the dosing interval for *S. pyogenes*. Finally, in amoxicillin simulations, where clavulanic acid was not present to protect amoxicillin, negligible values were obtained for both β-lactamase producing strains, and *f*T_>MIC_ decreased when comparing bacteria-free vs. mixed inocula simulations from 43.2% to 17.7% (*S. pneumoniae*) and from 99.9% to 24.9% (*S. pyogenes*) in the case of the gram-positive strains.

### Fitness in antibiotic-free simulations

**Figure 2:**
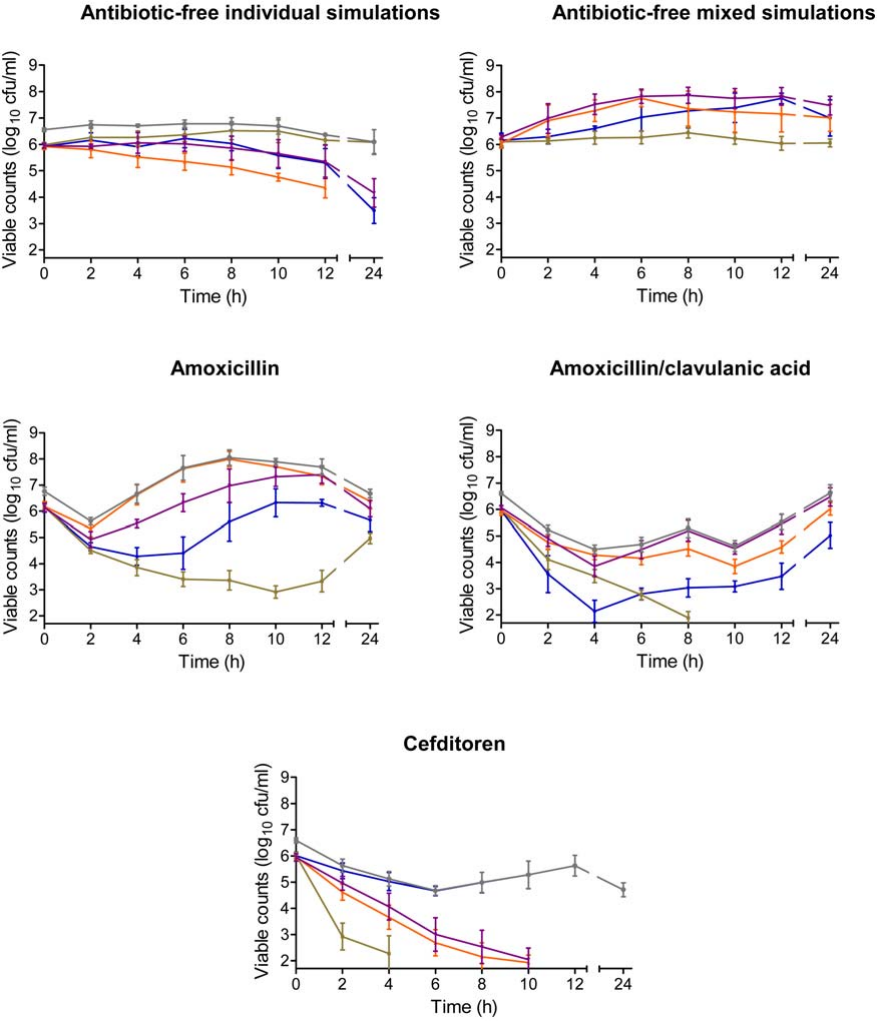
Evolution of inocula over time under experimental conditions. Colony counts over 24 h in antibiotic-free individual simulations, antibiotic-free mixed simulations and in simulations with study drugs (grey: total population, blue: *S. pneumoniae*, green: *S. pyogenes*, orange: BL^+^, purple: BLPACR).

Viability in THSB over time in individual and mixed simulations without antibiotics is shown in Figure 2. In individual cultures mean colony counts increased from time 0 to 12 and 24 h in ≥1 log_10_ cfu/ml for all strains except *S. pyogenes* where similar colony counts were found at times 0, 12 and 24 h (≈6.05 log_10_ cfu/ml).

The situation was completely different in simulations with the mixed inocula. Mean log_10_ colony counts of the global mixed inocula were maintained over time (6.55 log_10_ at time 0, 6.37 log_10_ at 12 h and 6.10 log_10_ at 24 h), but the distribution of the bacterial population highly varied when considering the percentage of cfu/ml of each strain over 24 h. At time 0 each strain accounted for approx. 25% of the mixed inocula, and at 24 h up to 96.3% of the population corresponded to *S. pyogenes*, with small populations of the BLPACR strain (3.17%) and *S. pneumoniae* (0.50%), and colony counts below the detection limit for the BL^+^ strain.

### Simulations with antibiotics and mixed inocula

A reduction of 98.5% (≈2 log_10_ cfu/ml) in the initial global mixed inocula at 24 h was obtained with cefditoren, but no reductions were obtained with amoxicillin and amoxicillin/clavulanic acid.

The reduction provided by cefditoren was due to the disappearance of the *S. pyogenes* population at 6 h (*f*T_>MIC_ 79%), of *H. influenzae* populations at 12 h (with *f*T_>MIC_ 58%), and to 94% decrease in the initial *S. pneumoniae* population at 24 h (with *f*T_>MIC_ ≈25%).

In amoxicillin/clavulanic acid simulations the maintained counts of BL^+^ (*f*T_>MIC_ 32.8%), and the increase in 0.5 log_10_ of the BLPACR population (*f*T_>MIC_ of only 4.6%) were responsible for the absence of reduction in the initial global mixed inocula. *S. pneumoniae* decreased 0.94 log_10_ (*f*T_>MIC_ 32.8%) and *S. pyogenes* was not detectable from 10 h on (*f*T_>MIC_ 99.9%).

In amoxicillin simulations, the maintenance or slight increase in *H. influenzae* strains (*f*T_>MIC_ 0%) produced the absence of reductions in the initial global mixed inocula at 24 h. *S. pneumoniae* decreased only 0.49 log_10_ and this could be related to the decrease in *f*T_>MIC_ from 43.2% in bacteria-free simulations to 17.7% in mixed inocula simulations due to β-lactamase production and amoxicillin inactivation as shown in Table 1 (significantly lower amoxicillin concentrations in mixed inocula vs. bacteria-free simulations). This inactivation also affected the activity against *S. pyogenes*, and the amoxicillin regimen was the only one that was not able to eradicate this strain (although producing 1.2 log_10_ reduction), due to the reduction in *f*T_>MIC_ from 99.9% to 24.9% when comparing bacteria-free vs. mixed inocula simulations.

### β-lactamase activity

Table 3 shows β-lactamase activity over 24 h in the simulations carried out with the study drugs. No differences were found between the three antibiotic regimens until 6 h when differences between amoxicillin and amoxicillin/clavulanic acid simulations were significant. At 10 and 12 h significant higher β-lactamase activity was found in amoxicillin than in amoxicillin/clavulanic acid simulations, and this could be related with the different population of the β-lactamase producing strains found in both simulations at these timepoints: approx. 7.5 log_10_ in amoxicillin and approx. 4–5 log_10_ in amoxicillin/clavulanic acid simulations. In cefditoren simulations, at 10 h and 12 h, the β-lactamase activity determined was similar to the one in amoxicillin simulations. Cefditoren was highly active against the two *H. influenzae* strains, and the lysis of these organisms (colony counts ≤100 colonies at these timepoints) probably produced release of the enzyme responsible for the high β-lactamase activity determined.

**Table 3:** β-lactamase activity.

Time	Cefditoren	Amoxicillin	Amoxicillin/clavulanic acid
0	0.21±0.00	0.21±0.00	0.24±0.00
Tmax	0.11±0.10	0.06±0.03	0.02±0.03
2	0.06±0.07	0.09±0.05	0.00±0.00
4	0.18±0.13	0.17±0.03	0.05±0.00
6	0.21±0.08	0.36±0.11	0.10±0.00^b^
8	0.31±0.11	0.23±0.04	0.14±0.00
10	0.23±0.11	0.17±0.06	0.02±0.02^c^
12	0.24±0.05	0.30±0.02	0.01±0.01^c^
24	0.05±0.02^d^	0.19±0.06	0.20±0.01

β-lactamase activity (absorbance units) in simulations of bid 400 mg cefditoren, tid 875 mg amoxicillin, and tid 875/125 mg amoxicillin/clavulanic acid regimens.

a Tmax: 2.8 h for cefditoren, 1.5 h for clavulanic acid and amoxicillin.

b p<0.01 amoxicillin/clavulanic acid vs. amoxicillin.

c p<0.01 amoxicillin/clavulanic acid vs. amoxicillin and cefditoren.

d p<0.01 cefditoren vs. amoxicillin and amoxicillin/clavulanic acid.

At 24 h significantly lower β-lactamase activity (0.05 absorbance units) was found in cefditoren simulations (*H. influenzae* colony counts below the limit of detection) vs. amoxicillin with or without clavulanic acid (0.20 absorbance units, a value similar to time 0) simulations where colony counts were higher than the initial inocula.

## Discussion

Ecology and resistance in human microbiota are related phenomena since there is some evidence that bacterial fitness decreases, at least at short term, due to resistance [29]. In compartment models the fitness of a bacterial strain is directly proportional to its ability to compete with other strains and inversely proportional to its clearance [30]. If there is heterogeneity in fitness, those strains with higher fitness are anticipated or eventually prevailed [30]. Dynamics of the different subpopulations in an antibiotic-free environment are the baseline that antibiotic treatments can alter by interfering with bacterial fitness (including colonisation and transmission), since resistance can be associated with a decrease in fitness [31].

In this study we tried to simulate a nasopharyngeal niche by means of a mixed inocula of common respiratory isolates showing classical (β-lactamase production in *H. influenzae* and penicillin resistance in *S. pneumoniae*) and emergent but increasing (concomitant *ftsI* gene mutation in the BLPACR strain) resistance phenotypes. Despite up to 80% of healthy persons carry *H. influenzae* [4], with multiple strains in 50% positive samples [5] and although there is an increasing isolation rate of BLPACR [11], the use of strains with different resistance phenotypes may be a limitation of the study. However resistance was needed as a marker to show differences between the three antibiotics used with different behaviour (susceptibility, protection by an inhibitor or resistance) in the presence of β-lactamases. Another limitation of the study may be the exposition to antibiotics of mixed inocula with identical proportions of the different strains at time 0. In a pathological situation, the inoculum of *S. pyogenes* may be higher than that of the other microorganisms, as occurred in this study in antibiotic-free simulations: *S. pyogenes* was the dominant population at 24 h (accounting for 96.3%) together with a small population of β-lactamase producing *H. influenzae* strain (3.17%). β-lactamase producing bacteria are frequently found in patients with recurrent tonsillitis, where the exposure to antibiotics in general and to β-lactams in particular select them in nasopharyngeal flora [14]. This has lead to the concept of co-pathogenicity (protection of *S. pyogenes* susceptible to penicillin by colocalized bacteria resistant to penicillin due to β-lactamase production) [32]–[34]. Co-pathogenicity has been hypothesised by some authors to be responsible of penicillin treatment failures in the treatment of group A β-hemolytic streptococci pharyngitis due to β-lactamase producing organisms in the pharynx as *H. influenzae* or *Moraxella catarrhalis* [34], but criticised by others [35], [36] on the basis that if there is a clinical difference (that may be dubious) between penicillin and cephalosporins, the question of how important is this difference, together with cost issues, remains. On the other side, in addition to the clinical goal of antimicrobial therapy that should be to eradicate the infecting pathogen, minimization of resistance selection in normal nasopharynx flora, and prevention of transmission of resistant clones from nasopharynx should also be taken into account.

Pharmacodynamic parameters as serum *f*T_>MIC_ for β-lactams can be related to bacterial eradication at the infection site, and the subsequent therapeutic outcome together with prevention of resistance [37] also in normal flora. This study explores the effects of serum drug profiles over 24 h of different β-lactam regimens on the natural evolution of the simulated niche. Although it may be argued that serum concentrations do not resemble nasopharynx concentrations, free-drug concentrations were used as an approximation despite the fact that protein binding of the highly bound cephalosporin may not highly influence bacterial killing [38]. In addition, in order to know the effects of β-lactamase production on antibiotic pharmacokinetics and its consequences on pharmacodynamic parameters predicting antibacterial activity, pharmacokinetic parameters were determined with concentrations measured in bacterial mixed inocula simulations and in bacteria-free simulations. The presence of the β-lactamase did not alter the concentrations of cefditoren (TEM-1 β-lactamase resistant) but highly modified those of amoxicillin over the entire dosing interval in amoxicillin tid regimen simulations, and in a lesser extent (from 4 h on) in amoxicillin/clavulanic acid tid regimen simulations. This led to pharmacokinetic parameters of amoxicillin significantly lower in mixed inocula simulations, with *f*T_>MIC_ values different if β-lactamase activity is considered or not.

As a result, the activity of cefditoren was not influenced by the presence of β-lactamase, and this cephalosporin eradicated *S. pyogenes* and co-pathogens as *H. influenzae*, and decreased *S. pneumoniae* population in 94%. In the case of the tid regimen of amoxicillin/clavulanic acid, the β-lactamase production by the BLPACR strain (resistant to amoxicillin/clavulanic acid: *f*T_>MIC_ of only 4.6%) protected the BL^+^ *H. influenzae* that although susceptible to amoxicillin/clavulanic acid (MIC = 2 µg/ml; *f*T_>MIC_ 32.8%), was not eradicated or even diminished. The same occurred with *S. pneumoniae* (MIC = 2 µg/ml; *f*T_>MIC_ 32.8%) that was only reduced in <1 log_10_. However the presence of the β-lactamase did not avoid eradication of *S. pyogenes* (MIC = 0.03 µg/ml; *f*T_>MIC_ 99.9%). On the contrary, in tid amoxicillin simulations, the maintenance and even increase in β-lactamase producing strains (*f*T_>MIC_ 0%) were able to protect not only *S. pneumoniae* (because a decrease in *f*T_>MIC_ from 43.2% to 17.7% in bacteria-free vs. mixed inocula simulations) but also *S. pyogenes* (decrease in *f*T_>MIC_ from 99.9% to 24.9%) from eradication.

The bacteriological failure rates of penicillins increased from 2 to 10% in the early 70's, but beginning in the late 70's, penicillin bacteriological failure rates increased to 30% in 2000's [39], maybe as the prevalence of β-lactamase production increased. Despite different causes for treatment failure (lack of compliance, reexposure, eradication of pharyngeal flora, bacterial fitness), and contradictory reports on the effects of co-pathogenicity [39], [40], the results of this study reinforces the concept of “indirect pathogen” since β-lactamase production in mixed inocula (resembling pharyngeal flora) decreased amoxicillin concentrations and pharmacokinetic parameters against *S. pneumoniae* or *S. pyogenes*, when clavulanic acid was not present, in such a magnitude that adequate values were not obtained and eradication was precluded. The presence of clavulanic acid along a tid regimen countered this effect of indirect pathogenicity by protecting amoxicillin from β-lactamase degradation, and a very susceptible strain as *S. pyogenes* was eradicated. However since the β-lactamase was produced by a strain resistant to amoxicillin/clavulanic acid (as the BLPACR strain), clavulanic acid was not able to protect amoxicillin from degradation in such an extent to allow its bactericidal activity against strains with MICs in the limit of susceptibility, as the *H. influenzae* BL^+^ and *S. pneumoniae* (MICs of 2 µg/ml) that were protected from eradication, thus selecting them. The quality of TEM β-lactamase resistance of cefditoren avoided this co-pathogenicity effect of *H. influenzae* β-lactamase production.

Although this study is only an approach of what could occur in the human nasopharynx, the results suggest that, at least in vitro, the presence of β-lactamase producing microorganisms may protect other microorganisms present in the niche. This effect of “indirect pathogen” or co-pathogenicity seems to be gradual since β-lactamase inhibitors (as calvulanic acid in tid regimens) countered it for strains very susceptible to amoxicillin as *S. pyogenes* but not for susceptible strains with amoxicillin MICs values in the limit of susceptibility as *S. pneumoniae*. These in vitro findings indicate a potential therapeutic advantage for β-lactamase resistant cephalosporins with high intrinsic activity against streptococci.
